# Glucose metabolism and function of CD4 ^+^ Tregs are regulated by the TLR8/mTOR signal in an environment of SKOV3 cell growth

**DOI:** 10.1002/cam4.6247

**Published:** 2023-06-14

**Authors:** Ming Wu, Xin Fu, Rui Xu, Shuna Liu, Rong Li, Juan Xu, Wenwen Shang, Xian Chen, Ting Wang, Fang Wang

**Affiliations:** ^1^ Department of Laboratory Medicine the First Affiliated Hospital of Nanjing Medical University Nanjing China; ^2^ Branch of National Clinical Research Center for Laboratory Medicine Nanjing China; ^3^ Department of Clinical Laboratory Children’s Hospital of Fudan University, National Children’s Medical Center Shanghai China; ^4^ Department of Anesthesiology Nanjing Drum Tower Hospital, the Affiliated Hospital of Nanjing University Medical School Nanjing China; ^5^ Department of Gynecology Women’s Hospital of Nanjing Medical University (Nanjing Maternity and Child Health Care Hospital) Nanjing China

**Keywords:** glucose metabolism, mammalian target of rapamycin, ovarian cancer, regulatory T‐cells, Toll‐like receptor 8

## Abstract

**Purpose:**

To investigate the role of mammalian target of rapamycin (mTOR) signal in Toll‐like receptor (TLR) 8‐mediated regulation of glucose metabolism and its effect on reversing immunosuppression in CD4^+^ regulatory T‐cells (Tregs) in ovarian cancer (OC).

**Methods:**

Fluorescence‐activated cell sorting was used to detect the expression levels of mTOR^+^ and 4E‐BP1^+^ cells in CD4^+^ Tregs. The prognosis and immune infiltration analysis of mTOR mRNA in OC were performed using the TIMER and Kaplan–Meier plotter database. Furthermore, real‐time polymerase chain reaction (RT‐PCR) and western blot (WB) were used to detect expression levels of glucose metabolism‐related genes and proteins in CD4^+^ Tregs. Glucose uptake and glycolysis levels were detected by colorimetry, while the effects of CD4^+^ Tregs on the proliferation of CD4^+^ T‐effector cells (Teffs) were evaluated by carboxyfluorescein diacetate succinimidyl ester (CFSE).

**Results:**

mTOR expression in CD4^+^ Tregs was significantly higher in patients with OC compared with controls and in CD4^+^ Tregs than in CD4^+^ Teffs in OC. Additionally, the expression level of mTOR mRNA was related to prognosis and immune infiltration levels in patients with OC. Blocking the mTOR signal resulted in downregulation of glucose metabolism in CD4^+^ Tregs. Simultaneous inhibition of the mTOR signal while activation of the TLR8 signal had a coordinated inhibitory effect on glucose metabolism and the immunosuppressive function of CD4^+^ Tregs. Furthermore, the mTOR signal played an essential role in TLR8‐mediated reversal of immunosuppressive function in CD4^+^ Tregs.

**Conclusion:**

These findings imply that activation of the TLR8 signal inhibits glucose metabolism in CD4^+^ Tregs by downregulating mTOR signaling, thereby reversing the immunosuppressive function of these cells in an OC cell growth environment.

## 1 INTRODUCTION

Ovarian cancer (OC) has the highest mortality rate among all gynecological malignancies. According to a report from 2022, there were 19,880 new cases of OC and 12,810 deaths related to it, with a survival rate of <40% after 5 years.^1^ Radical surgery combined with adjuvant chemotherapy is the primary treatment for OC currently. However, its therapeutic effect is still suboptimal.^2^ Immunotherapy holds promise as a treatment for OC because of its low toxicity, few side effects, and high precision, leading to a long‐lasting curative effect.^3^, ^4^ Targeting immunosuppression in the tumor microenvironment is an essential strategy in immunotherapy for cancer, where regulatory T‐cells (Tregs) play a crucial role in this approach to treatment.^5^, ^6^, ^7^, ^8^

The survival, growth, and functions of T cells are maintained by metabolism at the cellular level.^9^ The mammalian target of rapamycin (mTOR) signal transduction pathway plays a vital role in several cell processes such as growth, survival, proliferation, apoptosis, angiogenesis, and autophagy.^10^, ^11^ This pathway is commonly activated in tumors, and its downstream molecules, namely eIF4E‐binding protein 1 (4E‐BP1) and ribosome S6 protein kinase (P70S6K), control metabolism at the cellular level in the tumor microenvironment by altering the expression and/or activity of certain key metabolic enzymes. mTOR signaling can also be affected by metabolic changes, such as increased glucose or amino acid uptake.^12^, ^13^

The Toll‐like receptor (TLR) family comprises a group of pattern recognition receptors that identify various pathogen‐related molecular patterns and serve as the primary bridge between innate immunity and specific immunity.^14^, ^15^ The Toll‐like receptor can regulate the differentiation and function of Tregs directly or indirectly.^16^, ^17^, ^18^, ^19^ Among these TLR signals, the human TLR8 signal is highly expressed on human Tregs. When combined with its ligand, it can directly reverse the immunosuppressive function of both normal and tumor‐derived Tregs.^14^, ^20^ However, the exact mechanism by which TLR8 reverses the immunosuppressive function of Tregs in the tumor microenvironment of OC remains unclear.

Our previous study suggested that CD4^+^ Tregs had more active glucose metabolism than CD4^+^ T‐effector cells (Teffs) in the peripheral blood of patients with OC. Additionally, glucose metabolism was more active in patients with OC than in those with benign ovarian tumors (BOT) or healthy controls (HC).^21^ However, the exact role of the mTOR signal in reversing the immunosuppressive effect of OC‐derived Tregs remains unclear. Therefore, the objective of this study was to investigate the role of the mTOR signal in TLR8‐mediated regulation of glucose metabolism and reversal of the immunosuppressive function of CD4^+^ Tregs in OC.

## 2 MATERIALS AND METHODS

### 2.1 Patients and specimens

The study included 10 untreated patients with OC who were admitted to the First Affiliated Hospital of Nanjing Medical University from June 2020 to January 2021 (Table 1). At the same time, 10 BOT patients and 10 HC volunteers were selected as controls. All patients had a confirmed diagnosis of OC through cytology or histology and had not received surgery, chemotherapy, or another immunotherapy treatments. Patients with a history of another malignancy, major infectious disease, serious medical conditions such as heart failure, or diabetes were excluded. The study was approved by the Ethics Committee of the First Affiliated Hospital of Nanjing Medical University (2017‐SRFA‐064). All patients and control subjects provided written informed consent before taking part in this study.

**TABLE 1:** Characteristic features of ovarian cancer patients (*n* = 10).

Variables	Patients, *n* (%)
Age (years)	
<50	3 (30)
≥ 50	7 (70)
Histology	
Serous	5 (50)
Mucinous	1 (10)
Endometrioid	3 (30)
Clear cell carcinoma	1 (10)
FIGO stage	
I‐II	5 (50)
III‐IV	5 (50)
Lymph node metastasis	
No	6 (60)
Yes	4 (40)

### 2.2 Data acquisition and preprocessing

The study involved the analysis of single‐cell RNA sequencing data and mRNA RNA‐seq data from human OC samples. The single‐cell RNA sequencing data of seven human primary OC samples were obtained from the gene expression omnibus (GEO) database (GSE184880, http://www.ncbi.nlm.nih.gov/geo/).^22^ Data preprocessing and analysis were performed using the scanpy package. To analyze the association between mTOR mRNA and immune cell infiltration level, the Tumor Immune Estimation Resource 2 (TIMER 2) database (http://timer.cistrome.org/) was used. Additionally, the correlation between mTOR mRNA and prognosis of patients with OC was analyzed using the Kaplan–Meier Plotter database (http://kmplot.com/analysis/index.php?p=background).

### 2.3 Flow cytometry

Freshly isolated peripheral blood mononuclear cells (PBMCs, 1 × 10^6^) were washed and incubated with fluorophore‐conjugated monoclonal antibodies (anti‐CD4‐fluorescein isothiocyanate, anti‐CD25‐allophycocyanin, and anti‐CD127‐BV421) for 30 min in the dark at room temperature. For intracellular staining, 1 × 10^6^ PBMCs were washed, fixed, permeabilized, and stained with anti‐mTOR‐phycoerythrin, following the manufacturer's protocol. To detect intranuclear transcription factor, 1 × 10^6^ cells were washed, fixed, permeabilized, and stained with 4E‐BP1 rabbit monoclonal antibody and anti‐rabbit immunoglobulin G (H + L) F(ab')2 Fragment (phycoerythrin conjugate) according to the manufacturer's protocol. Fluorescence data were collected on a Fluorescence‐activated cell sorting (FACS) Aria II cell sorter (BD Biosciences) and analyzed using FlowJo software (Tree Star).

### 2.4 Cell sorting and culture of cell lines

CD4^+^ Tregs and Teffs were isolated from PBMCs using FACS following staining with fluorescein isothiocyanate‐anti‐CD4, allophycocyanin‐anti‐CD25, phycoerythrin‐anti‐CD127 (all from BD Biosciences) using the FACS Aria II cell sorter. The purity of the sorted cells was >95%.

The human ovarian serous adenocarcinoma cell line (SKOV3) was purchased from the American Type Culture Collection and cultured in McCoy's 5A medium (Invitrogen) supplemented with 10% fetal bovine serum at 37°C in 5% CO_2_.

### 2.5 Amplification of CD4^+^ Tregs and Teffs in vitro

The CD4^+^ Tregs and Teffs were washed and resuspended in X‐Vivo 15 medium (Lonza) supplemented with 5% human AB serum (Gemini), to which 500 U/mL of interleukin‐2 (Peprotech) was added. The cells were then seeded at a concentration of 1 × 10^6^ cells per well into 96‐well round‐bottomed plates containing 20 μL of anti‐CD3/CD28‐coated microbeads (Invitrogen) per well. When the cell populations had doubled, the suspension was divided equally between two wells and cultured for 2 weeks.

### 2.6 Coculture experiments

CD4^+^ Tregs and Teffs (1.6 × 10^6^/mL) amplified from healthy donor's peripheral blood were cultured in a transwell chamber containing X‐Vivo 15 medium with SKOV3 cells (4 × 10^5^/mL) in 24‐well plates at 37°C in 5% CO_2_ atmosphere. After 3 days, T‐cells were harvested for subsequent experiments.

### 2.7 Isolation of RNA and quantitative real‐time polymerase chain reaction

Total RNA was extracted from CD4^+^ Tregs and Teffs using the RNeasy Micro kit (Qiagen) and then reverse transcribed into complementary DNA with Prime Script RT Master Mix (Takara) following the manufacturer's instructions. Genes related to glucose metabolism and mTOR signal mRNA levels were quantified using TB Green and a 7500 Real‐Time PCR system (Applied Biosystems, Life Technologies). The primer sequences are shown in in Table 2. The relative expression of these target genes normalized by β‐actin was calculated as 2^−ΔCt^.

**TABLE 2:** Primer sequences used in polymerase chain reaction.

Gene name	Forward primer (5′→3′)	Reverse primer (5′→3′)
β‐actin	GAGCTACGAGCTGCCTGACG	GTAGTTTCGTGGATGCCACAG
HIF‐1α	CCATTAGAAAGCAGTTCCGC	TGGGTAGGAGATGGAGATGC
Glut1	TTGGCTCCGGTATCGTCAAC	GCCAGGACCCACTTCAAAGA
GPI	AGGCTGCTGCCACATAAGGT	AGCGTCGTGAGAGGTCACTTG
ENO1	TCATCAATGGCGGTTCTCA	TTCCCAATAGCAGTCTTCAGC
PKM2	GCCGCCTGGACATTGACTC	CCATGAGAGAAATTCAGCCGAG
LDHα	CCAGCGTAACGTGAACATCTT	CCCATTAGGTAACGGAATCG
TPI	AGGCATGTCTTTGGGGAGTC	AGTCCTTCACGTTATCTGCGA
Glut3	GCTCTCTGGGATCAATGCTGTGT	CTTCCTGCCCTTTCCACCAGA

### 2.8 Western blotting analysis

After various treatments, CD4^+^ Tregs and Teffs were washed and harvested. Whole‐cell lysates were prepared from T‐cells for western blot (WB) analyses. Western blot were developed with Chemiluminescent Substrate (Thermo Fisher Scientific). The rabbit polyclonal antibodies used for WB were rabbit anti‐LDH‐α, anti‐Glut1, anti‐PKM2, anti‐HIF‐1α, anti‐β‐actin, and anti‐GAPDH (Cell Signaling Technology).

### 2.9 Glucose uptake and glycolysis assay

CD4^+^ Tregs and Teffs were washed, resuspended, and starved overnight in serum‐free medium. The following morning, glucose uptake by the cells was measured using a Biovision glucose uptake kit (Biovision) following the manufacturer's instructions.

After various treatments, the cells supernatants were collected. Glycolysis levels were determined using a glycolysis cell‐based assay kit (Cayman Chemical) following the manufacturer's instructions.

### 2.10 Assay for proliferation of CD4^+^ Teffs

CD4^+^ Tregs were treated with the TLR8 ligand (ssRNA40), mTOR signal inhibitor (rapamycin), and mTOR signaling pathway activator (MHY1485) to create an experimental group. Cells that did not receive this treatment were used as a control group. Next, we cocultured 1 × 10^6^ CD4^+^ Tregs from each of these groups with CD4^+^ Teffs at a ratio of 1:1 in round‐bottomed 96‐well plates containing anti‐CD3 and anti‐CD28 antibody (1 μg/mL). The CD4^+^ Teffs were stained with carboxyfluorescein succinimidyl ester (CFSE) dye (2 μM, Invitrogen) following the manufacturer's instructions. CD4^+^ Teffs (1 × 10^5^) were added to a well without anti‐CD3 and anti‐CD28 antibody as the parent peak. After 96 h of culture, Teff proliferation levels were determined using FACS.

### 2.11 Statistical analysis

These data are presented as the mean ± SEM. Differences between two groups were compared using the student's *t* test or the nonparametric Mann–Whitney *U* test. All statistical analyses were conducted using the Statistical Package for the Social Sciences software version 22.0 (IBM Corp). A *p <* 0.05 was considered statistically significant.

## 3 RESULTS

### 3.1 mTOR expression was increased in CD4^+^ Tregs and higher than that in CD4^+^ Teffs in patients with OC

**FIGURE 1:**
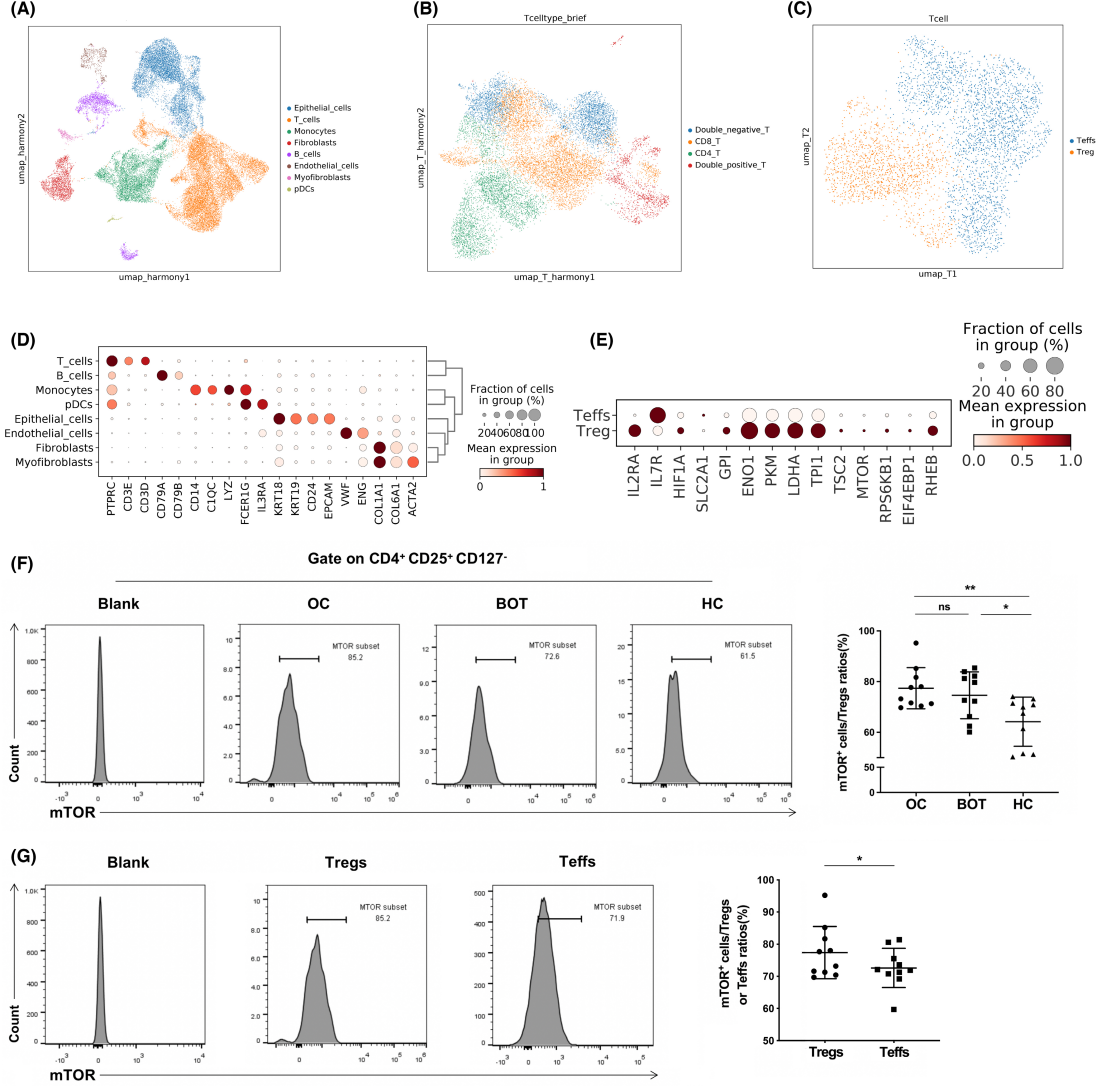
Expression level of mTOR in CD4^+^ Tregs and Teffs. (A–C) The UMAP plot demonstrates the main cell types in ovarian cancer tissues according to the composition of the marker genes. (D, E) Dot plots showing the expression levels of specific marker genes in each cell type based on results of single‐cell sequencing. The size of dots indicates the proportion of cells expressing the particular marker gene. The spectrum of color represents the mean expression levels of the marker genes. (F) The expression level of mTOR factor in CD4^+^ Tregs of human ovarian cancer, benign ovarian cancer and healthy control peripheral blood, as detected via flow cytometry (*n* = 10). Left images are the representative flow cytometry analysis and plots are gate on CD4^+^CD25^+^CD127^−^. Right bar diagram summarizes the expression ratios as the mean ± SEM; *p < 0.05, **p < 0.01. (G) Analysis of mTOR factor in CD4^+^ Tregs and Teffs of OC and plots are gate on CD4^+^CD25^+^CD127^−^ or CD4^+^CD25^−^CD127^+^ via flow cytometry (*n* = 10). Left images are the representative flow cytometric analysis and right bar diagram shows the proportions of mTOR in CD4^+^ Tregs and Teffs. Data are displayed as mean ± SEM; *p < 0.05.

We analyzed single‐cell RNA sequencing datasets from seven patients with OC. Cell visualization was performed using UMAP approaches, as shown in Figure 1A–C. Furthermore, Figure 1D illustrated the identification of ecosystematic cell types, such as T‐cells (marked by CD3D and CD3E), epithelial cells (marked by KRT18, EPCAM, CD24, and KRT19), and monocytes (CD14 and C1QA). After classifying and annotating cells, we analyzed expression levels of mTOR signal and glucose metabolism related genes in CD4^+^ Tregs (CD4^+^CD25^+^CD127^−^) and CD4^+^ Teffs (CD4^+^CD25^−^CD127^+^) in OC tissues. As shown in Figure 1E, expression levels of these genes in CD4^+^ Tregs were higher than those in Teffs.

We also evaluated mTOR factor expression level in CD4^+^ Tregs and Teffs in peripheral blood. CD4^+^ Tregs in peripheral blood were identified as CD4^+^CD25^+^CD127^−^ T‐cells, and CD4^+^ Teffs were identified as CD4^+^CD25^−^CD127^+^ T‐cells using FACS. The expression level of mTOR in CD4^+^ Tregs was higher in patients with OC than in HC but was not significantly different from that in patients with BOT (77.4% ± 8.12% vs. 64.19% ± 9.7% and 77.4% ± 8.12% vs. 74.6% ± 9.25%, respectively, *p <* 0.01 and *p >* 0.05; Figure 1F). Furthermore, mTOR expression in peripheral blood of patients with OC was significantly higher in CD4^+^ Tregs than in CD4^+^ Teffs (77.4% ± 8.12% vs. 72.61% ± 6.1%, *p <* 0.05; Figure 1G).

### 3.2 Prognosis and immune infiltration of mTOR mRNA expression in patients with OC

**FIGURE 2:**
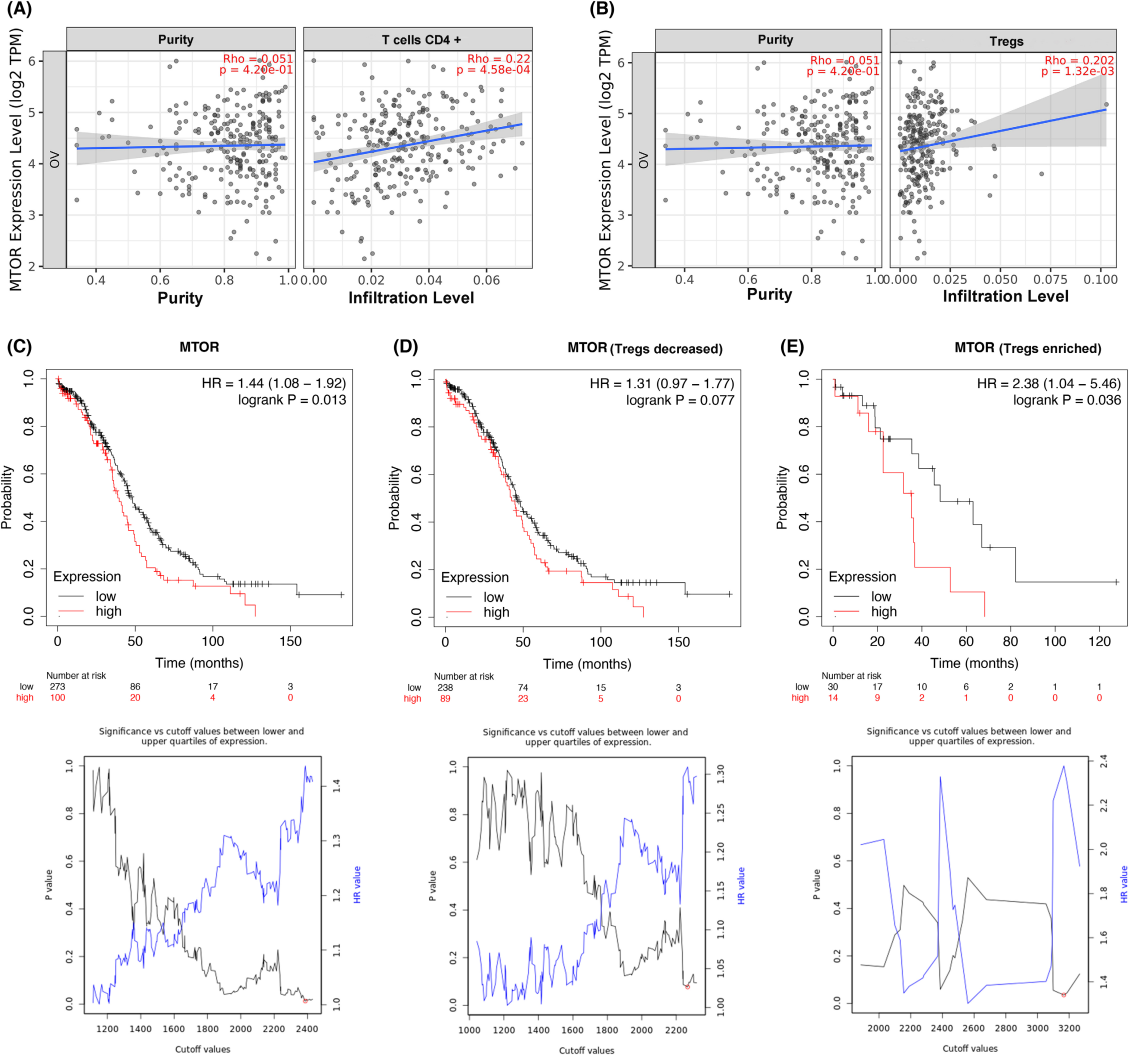
Immune infiltration and prognosis of mTOR gene expression in patients with ovarian cancer. (A,B) The expression level of mTOR mRNA was positively correlated with CD4^+^ T‐cells and Tregs infiltration level after purity adjustment in ovarian cancer tissues according to TIMER database (*n* = 303). (C) The relationship between mTOR mRNA and overall survival in patients with ovarian cancer (*n* = 374). (D, E) The relationship between mTOR mRNA and overall survival in ovarian cancer patients with different Tregs infiltration status (*n* = 374).

TIMER and the Kaplan–Meier plotter database were used for clinical analysis of mTOR gene status in patients with OC. We found a positive correlation between mTOR mRNA expression levels and infiltration levels of CD4^+^ T cells and Tregs in OC patients (*p =* 4.58E‐04, *ρ =* 0.22, *p =* 1.32E‐03, *ρ =* 0.202; Figure 2A,B). Furthermore, high mTOR mRNA expression was associated with poor prognosis in OC patients (hazard ratio [HR]: 1.44, *p =* 0.013, Figure 2C). We then analyzed the relationship between mTOR mRNA and patient prognosis based on Treg infiltration status. We found that high mTOR mRNA expression was related to a poor prognosis when the infiltration level of Tregs was high but not when it was low (HR: 2.38, *p =* 0.036 vs. HR: 1.31, *p =* 0.077; Figure 2D,E).

### 3.3 Activation of the TLR8 signal downregulated mTOR expression in CD4^+^ Tregs in the environment of OC cell growth

**FIGURE 3:**
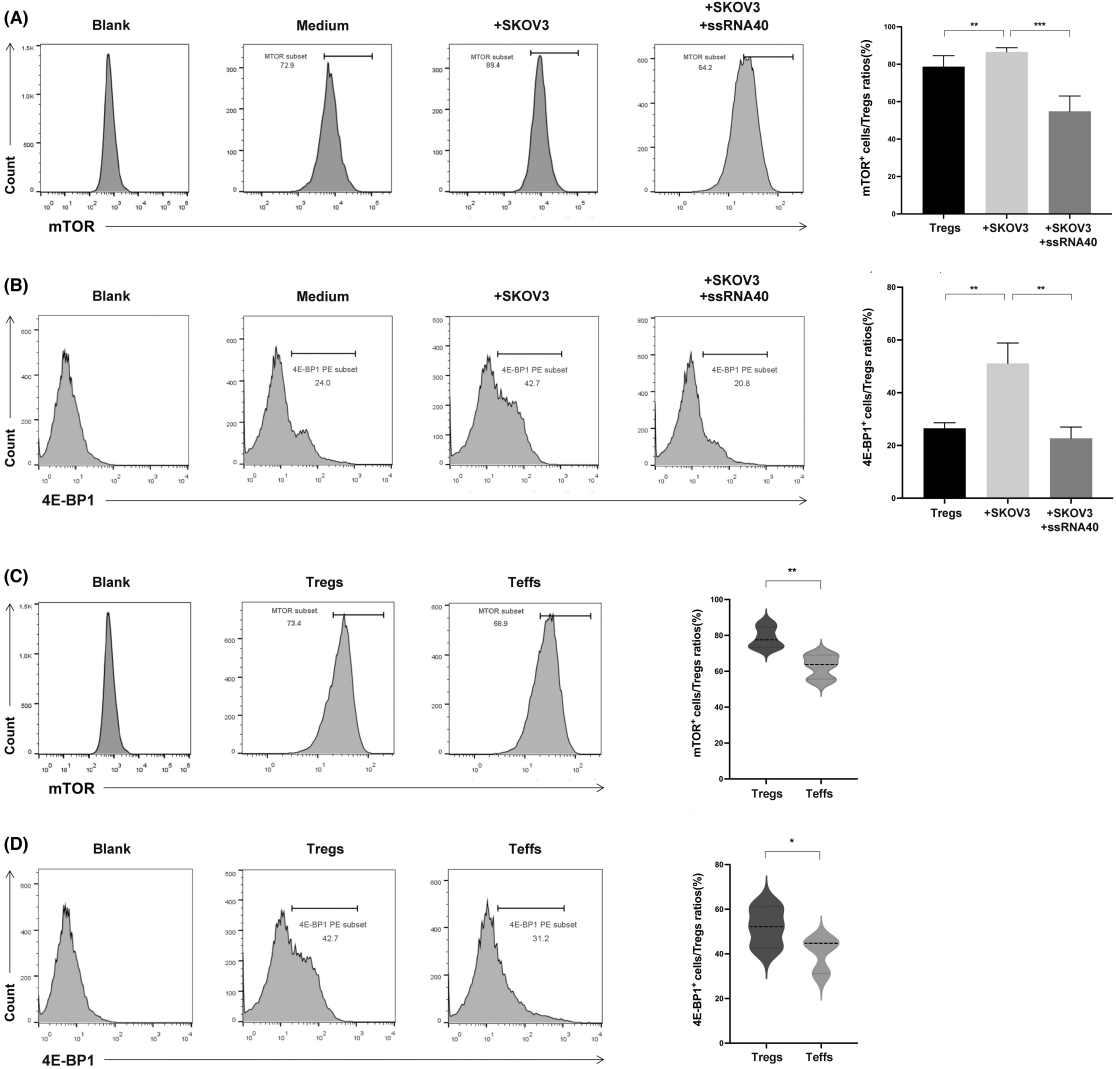
Expression levels of mTOR and 4E‐BP1 factors in CD4^+^ Tregs and Teffs (*n* = 6). (A, B) The expression levels of mTOR and 4E‐BP1 factors in CD4^+^ Tregs in different groups. Left images are the representative flow cytometric analysis. Right bar diagram shows the proportions of mTOR and 4E‐BP1in CD4^+^ Tregs and Teffs. Data are displayed as mean ± SEM; **p < 0.01, ***p < 0.001. (C, D) The expression levels of mTOR and 4E‐BP1 factors in CD4^+^ Tregs and Teffs in the SKOV3 growth environment. Left images are the representative flow cytometric analysis, and right bar diagram summarizes the expression ratios as the mean ± SEM, *p < 0.05, **p < 0.01.

4E‐BP1 is a downstream molecule in the mTOR signal and controls cell proliferation and function. We compared mTOR and 4E‐BP1 expression levels in CD4^+^ Tregs cocultured with SKOV3 cells to those of Tregs that were not cocultured with SKOV3 cells and found that they were significantly higher in the former group (86.55% ± 2.08% vs. 78.73% ± 5.30%, *p <* 0.01 and 51.08% ± 6.70% vs. 26.50% ± 1.84%, *p <* 0.01, respectively; Figure 3A,B). We also compared mTOR and 4E‐BP1 expression levels between CD4^+^ Tregs and CD4^+^ Teffs in the SKOV3 growth environment and found that they were higher in CD4^+^ Tregs than in CD4^+^ Teffs (78.73% ± 5.30% vs. 62.75% ± 5.79%, *p <* 0.01 and 42.20% ± 6.46% vs. 26.50% ± 1.84%, *p <* 0.05, respectively; Figure 3C,D). Furthermore, we examined mTOR and 4E‐BP1 expression levels in CD4^+^ Tregs following the activation of the TLR8 signal and found them to be downregulated (78.73% ± 5.30% vs. 54.83% ± 7.47%, *p <* 0.01 and 26.50% ± 1.84% vs. 22.70% ± 3.71%, *p <* 0.01, respectively; Figure 3A,B).

### 3.4 Blocking of the mTOR signal pathway downregulated glucose metabolism in CD4^+^ Tregs

**FIGURE 4:**
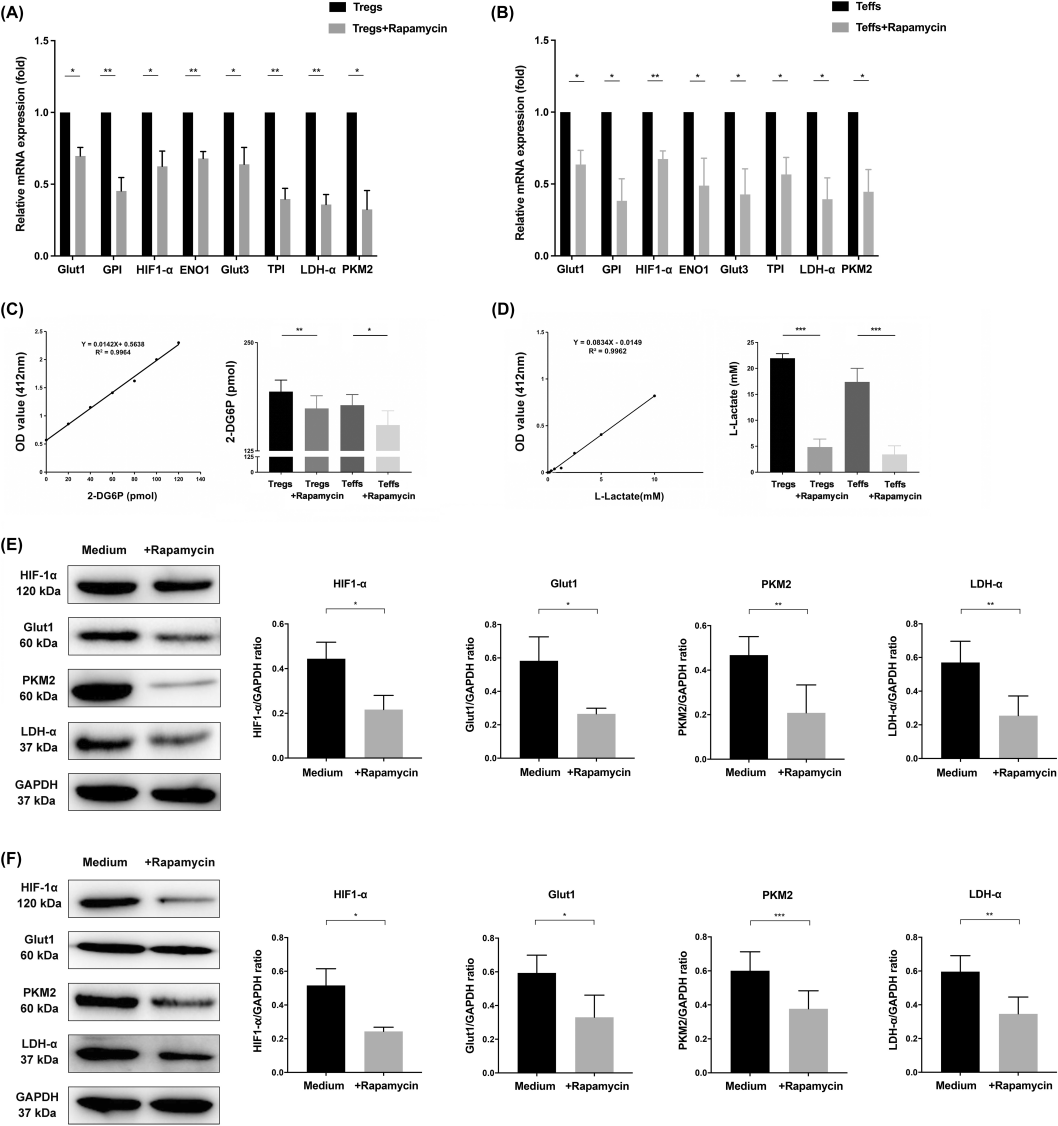
mTOR signaling pathway can regulate glucose metabolism level of CD4^+^ Tregs. (A, B) Expression levels of genes related to glucose metabolism (Glut1, GPI, HIF‐1α, Eno1, Glut3, TPI, LDH‐α, PKM2) in CD4^+^ Tregs and Teffs detected via quantitative real‐time PCR. Expression levels of each gene were normalized to β‐actin expression level and adjusted to the levels in CD4^+^ Tregs and Teffs not treated with rapamycin (served as 1). Data shown are mean ± SEM; *p < 0.05, **p < 0.01. (C) The levels of glucose uptake of CD4^+^ Tregs and CD4^+^ Teffs via colorimetry (*n* = 3). The left image is standard curve of glucose uptake, and the right is the 2‐DG6P level converted from the OD value according to the standard curve. Data shown are mean ± SEM; *p < 0.05, **p < 0.01. (D) The levels of glycolysis of CD4^+^ Tregs and CD4^+^ Teffs via colorimetry (*n* = 8). The left image is standard curve of glycolysis, and the right is the L‐Lactate level converted from the OD value according to the standard curve. Data shown are mean ± SEM; ***p < 0.001. (E,F) Expression levels of proteins related to glucose metabolism (Glut1, HIF‐1α, LDH‐α, PKM2) in CD4^+^ Tregs and Teffs detected by western blot. Five panels on the left showed the western blot analysis results. The panel on the right showed the protein expressions analyzed quantitatively and compared with GAPDH expression with a densitometer. Results shown in the histogram are mean ± SEM, *p < 0.05, **p < 0.01, ***p < 0.001. [Correction added on July 27, 2023 after first online publication. The figure 4E has been updated in this version.]

We have previously shown that TLR8‐mediated reversal of the immunosuppressive function of CD4^+^ Tregs is causally linked to glucose metabolism and that mTOR is a key metabolic signal transduction pathway.^21^, ^23^ Therefore, we explored the relationship between the mTOR signal and glucose metabolism in CD4^+^ Tregs in an environment of SKOV3 growth after treatment with rapamycin, a specific inhibitor of the mTOR signal. Expression levels of both genes and proteins (Figure 4A,E) related to glucose metabolism were significantly lower in cells treated with rapamycin than in control cells. Additionally, levels of glucose uptake (2‐DG6P value: 193.49 ± 13.28 vs. 174.05 ± 14.58, *p <* 0.01) and glycolysis (L‐lactate value: 21.97 ± 0.87 vs. 4.85 ± 1.54, *p <* 0.001) were downregulated in cells treated with rapamycin (Figure 4C,D). Rapamycin also downregulated expression levels of genes and proteins related to glucose metabolism in CD4^+^ Teffs (Figure 4B,F) as well as the levels of glucose uptake and glycolysis.

### 3.5 Inhibition of the mTOR signal and activation of the TLR8 signal had a synergistic inhibitory effect on glucose metabolism and immune function in CD4^+^ Tregs

**FIGURE 5:**
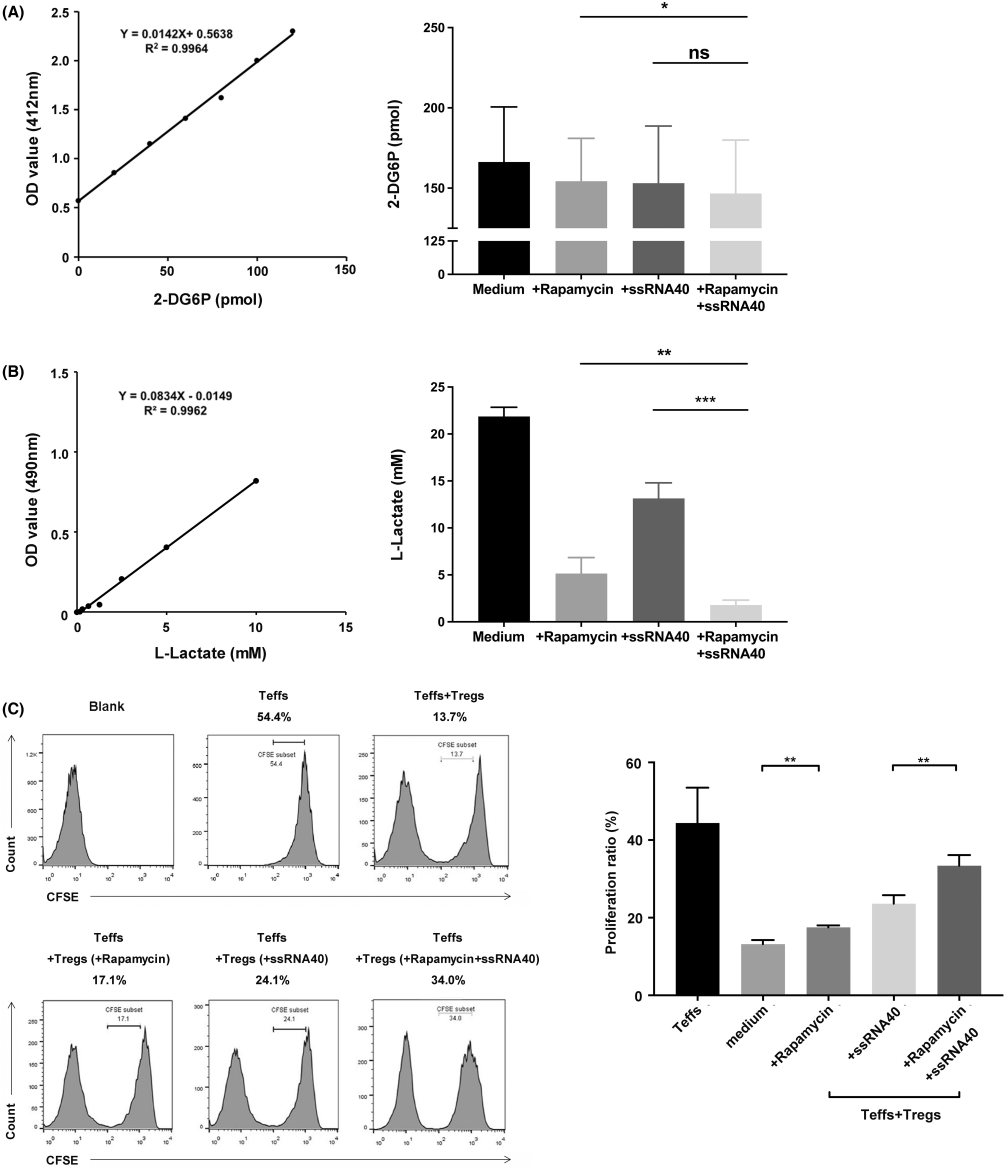
Inhibition of mTOR signal and activation of TLR8 signal has a synergistic inhibitory effect on the glucose metabolism and immune function of CD4^+^ Tregs. (A) The level of glucose uptake of CD4^+^ Tregs via colorimetry (*n* = 12). The left image is standard curve of glucose uptake, and the right is the 2‐DG6P level converted from the OD value according to the standard curve. Data shown are mean ± SEM; ns *p* > 0.05, *p < 0.05. (B) The level of glycolysis of CD4^+^ Tregs via colorimetry (*n* = 6). The left image is standard curve of glycolysis, and the right image is the L‐Lactate level converted from the OD value according to the standard curve. Data shown are mean ± SEM; **p < 0.01, ***p < 0.001. (C) The proliferation level of CD4^+^ Teffs co‐cultured with different groups of CD4^+^ Tregs. Left images were the representative flow cytometric analysis of CD4^+^ Teffs in different groups. Right bar diagram showed the proliferation level of CD4^+^ Teffs and results shown in the histogram are mean ± SEM; **p < 0.01.

To explore the relationship between the mTOR signal, TLR8 signal, glucose metabolism, and immunosuppressive function of CD4^+^ Tregs in SKOV3 growth environment, we blocked the mTOR signal in CD4^+^ Tregs and activated the TLR8 signal. We found that compared to treated with rapamycin or ssRNA40 alone, the glycolysis level of CD4^+^ Tregs treated with both rapamycin and ssRNA40 was significantly reduced (L‐lactate value: 5.14 ± 1.71 vs. 1.79 ± 0.53 and 13.15 ± 1.66 vs. 1.79 ± 0.53, *p <* 0.01 and *p <* 0.001, respectively; Figure 5A,B). Moreover, compared to the group treated with rapamycin alone, the glucose uptake level in CD4^+^ Tregs was significantly lower in the group that received combined treated with rapamycin and ssRNA40, but remained unchanged in the group that received ssRNA40 alone (2‐DG6P value: 154.28 ± 26.76 vs. 146.6 ± 33.33 and 153.07 ± 35.64 vs. 146.6 ± 33.33, *p <* 0.05 and *p >* 0.05, respectively). Thus, inhibition of the mTOR signal and activation of the TLR8 signal have a synergistic inhibitory effect on glucose metabolism in CD4^+^ Tregs.

We also examined the immunosuppressive function of CD4^+^ Tregs in the SKOV3 growth environment using the CFSE test and demonstrated that inhibition of the mTOR signal by rapamycin markedly reduced the ability of CD4^+^ Tregs to suppress proliferation of CD4^+^ Teffs. Furthermore, combined treatment with rapamycin and ssRNA40 significantly enhanced TLR8‐mediated reversal of CD4^+^ Treg suppression (Figure 5C).

### 3.6 mTOR signaling pathway had an important role in regulating TLR8‐mediated reversal of immunosuppression by CD4^+^ Tregs

**FIGURE 6:**
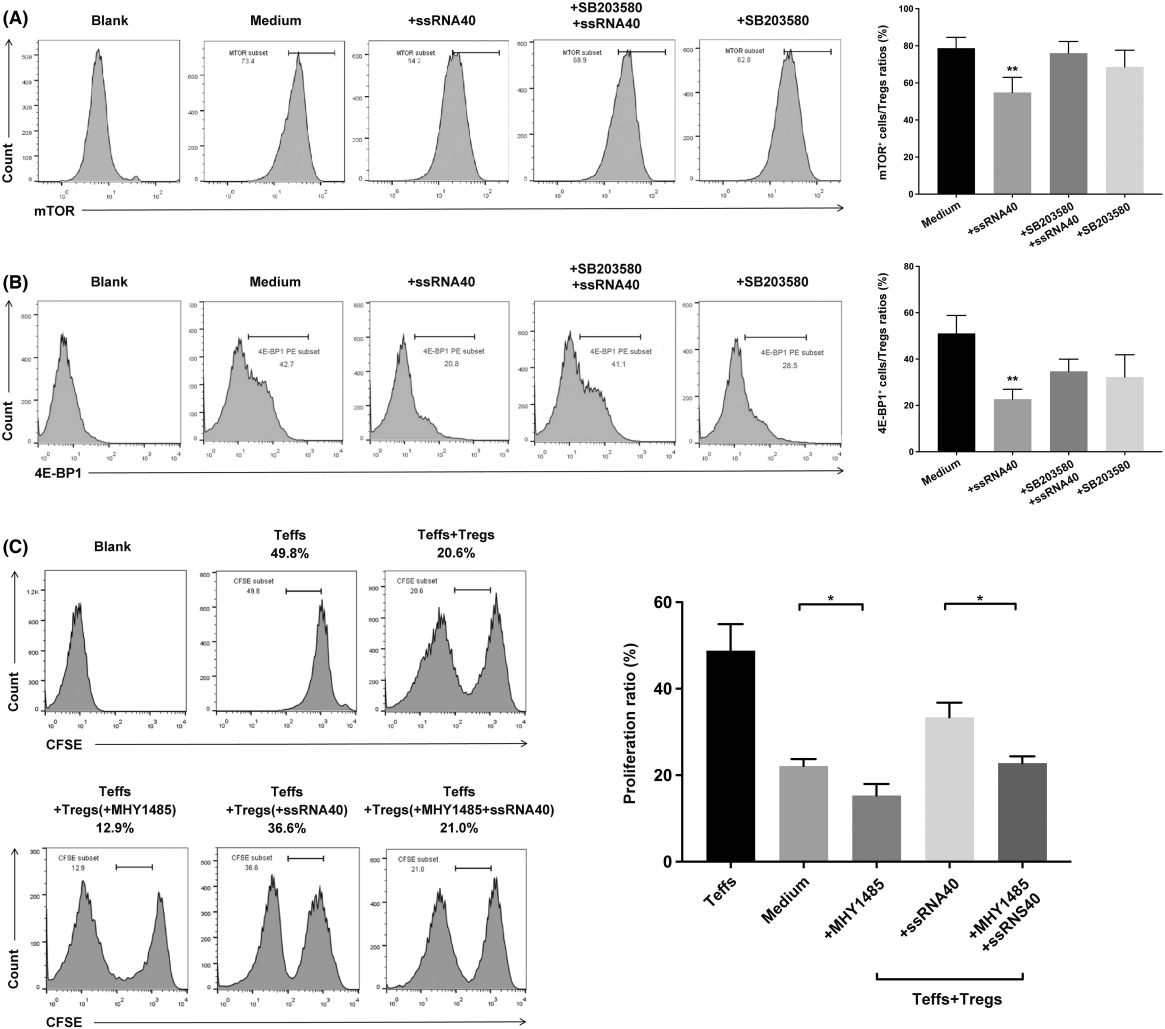
mTOR signaling pathway play an important role in regulating TLR8‐mediated reversal of CD4^+^ Tregs immunosuppressive. (A, B) The expression levels of mTOR and 4E‐BP1 in CD4^+^ Tregs treated with SB203580 or ssRNA40 in SKOV3 growth environment. Left images are the representative flow cytometric analysis. Right bar diagram shows the proportions of mTOR and 4E‐BP1in CD4^+^ Tregs and Teffs. Data are displayed as mean ± SEM; **p < 0.01. (C) The proliferation level of CD4^+^ Teffs co‐cultured with different groups of CD4^+^ Tregs. Left images are the representative flow cytometric analysis of CD4^+^ Teffs in different groups. Right bar diagram shows the proliferation ratio of CD4^+^ Teffs. Data were displayed as mean ± SEM, *p < 0.05.

In addition, we blocked p38 using SB203580 to investigate the involvement of p38 in regulating mTOR signal in CD4^+^ Tregs and to clarify the role of the mTOR signal in reversing the immunosuppressive function of CD4^+^ Tregs mediated by TLR8 signal in SKOV3 growth environment. We observed that blocking p38 signal in CD4^+^ Tregs reversed the downregulation of mTOR signal phosphorylation mediated by TLR8 (Figure 6A,B). Furthermore, we performed a functional rescue experiment and observed that activating the mTOR signal with MHY1485 prevented TLR8‐mediated reversal of the immunosuppressive effect of CD4^+^ Tregs and restored proliferation of CD4^+^ Teffs (Figure 6C).

## 4 DISCUSSION

An increasing number of studies have shown that mTOR serves as a downstream signal of several frequently mutated oncogenic pathways, such as the PI3K/AKT pathway and MAPK signaling pathway. This results in excessive activation of the mTOR signal in various tumors, thereby promoting tumor formation.^24^, ^25^ Our results suggest that mTOR is highly expressed in CD4^+^ Tregs at levels higher than those in CD4^+^ Teffs in the peripheral blood and tissues of patients with OC. Additionally, mTOR and Tregs are closely related and affect the prognosis of patients with OC. Blockade of the mTOR signal can downregulate glucose metabolism and immunosuppressive function in CD4^+^ Tregs in SKOV3 growth environment. However, inhibition of the mTOR signal also affects the glucose metabolism of CD4^+^ Teffs, indicating that the mTOR signal is also involved in regulating the glucose metabolism process of CD4^+^ Teffs. Therefore, the effect of reversing the immunosuppressive microenvironment only by mTOR signal inhibitors may not be optimal. In a word, it is crucial to find out the pathway downstream of mTOR signal that specifically regulates the immunosuppressive function of Tregs.

The TLR signal can directly or indirectly control the immunosuppressive function of Tregs. Among TLR members, several studies have demonstrated that TLR8 can specifically reverse immunosuppressive function of human Tregs.^18^ Interestingly, TLR8 signaling and Foxp3 can regulate the balance between rapamycin complex 1 (mTORC1) signal and glucose metabolism, thereby modulating the proliferation and immunosuppressive function of Tregs^19^ Our previous study also showed that TLR8 could reverse the suppression function of Tregs in the SKOV3 microenvironment, which was related to the mTOR signal.^21^ However, it was unclear whether there was a causal relationship between the two. Consistent with above research, our current results showed that activation of the TLR8 signal downregulated mTOR expression in CD4^+^ Tregs in SKOV3 growth environment, and mTOR signal played a crucial role in regulating TLR8‐mediated reversal of immunosuppression by CD4^+^ Tregs. Therefore, mTOR is also an important factor involved in the immunosuppressive microenvironment of OC induced by CD4^+^ Tregs. Moreover, the inhibition of glucose metabolism in CD4^+^ Tregs by rapamycin was more pronounced than that in CD4^+^ Teffs, and the inhibition of the mTOR signal and activation of the TLR8 signal had a synergistic inhibitory effect on glucose metabolism and immune function in CD4^+^ Tregs. Due to the nonspecificity of mTOR signal in regulating metabolism, one commonly used research method involves combining mTOR signal inhibitors with other reagents for antitumor treatment. The Saara Laukkanen team uncovered potent drug synergies between AKT/mTORC1 inhibitors and the general tyrosine kinase inhibitor dasatinib, and this combination effectively curbed T‐cell acute lymphoblastic leukemia (T‐ALL) growth in primary human T‐ALL.^26^

T‐cell responses are shaped by various signals from the tumor microenvironment (TME), including immune signals, growth factors, nutrients, oxygen, and bioactive metabolites. mTOR integrates these immune and environmental signals to direct T‐cell development, activation, and differentiation.^27^ This study expands on previous research to further explore the role of metabolic regulation mechanism in the immunosuppressive microenvironment of OC cells and to try to investigate whether TLR8/mTOR signal may be a potential target for tumor immunotherapy in the future. However, the current study has several limitations. Due to limited experimental conditions and time, we only conducted cytological experiments on one cell line. Therefore, it remains to be verified in vivo whether the TLR8/mTOR signal regulates the glucose metabolism of CD4^+^ Tregs to reverse their immunosuppressive function and ultimately enhanced antitumor effect. In addition, there are various TLR8 activators and mTOR inhibitors available, and different inhibitors may have different antitumor effects that require further investigation. Nonetheless, combined with clinical or animal experiments, targeting the metabolism of inhibitory immune cells through the TLR8/mTOR signal to suppress their function in OC immunotherapy is worth exploring further.

## 5 CONCLUSIONS

In summary, our present research deepens our understanding of the metabolic reprogramming of CD4^+^ Tregs and immune function in the presence of OC cells. The TLR8 signal, mTOR signal, and the metabolism and function of CD4^+^ Tregs are interrelated. Activation of the TLR8 signal can target and regulate the metabolism of CD4^+^ Tregs by downregulating the mTOR signal and the immune response at the cellular level through metabolic reprogramming. Although it remains a challenge to translate our research into clinical practice for antitumor therapy, these results may lead to the development of new immunotherapeutic strategies that can improve the anti‐tumor immune response in patients with OC.

## AUTHOR CONTRIBUTIONS

**Ming Wu:** Data curation (lead); formal analysis (lead); investigation (lead); writing – original draft (lead); writing – review and editing (lead). **Xin Fu:** Data curation (equal); writing – review and editing (equal). **Rui Xu:** Data curation (equal); investigation (equal); writing – review and editing (equal). **Shuna Liu:** Formal analysis (supporting); writing – review and editing (supporting). **Rong Li:** Resources (supporting); software (supporting). **Juan Xu:** Investigation (supporting); methodology (supporting). **Wenwen Shang:** Methodology (supporting); writing – review and editing (supporting). **Xian Chen:** Visualization (supporting); writing – review and editing (supporting). **Ting Wang:** Supervision (equal); validation (equal); writing – review and editing (equal). **Fang Wang:** Funding acquisition (equal); methodology (equal); validation (equal); visualization (equal); writing – review and editing (equal).

## FUNDING INFORMATION

This study was supported by grants from the National Natural Science Foundation of China (no. 82273199, 81772779), Natural Science Foundation of Jiangsu Province (no. BK20221417), Medical Talent of Empowering Medicine through Science and Education Program of Jiangsu Province (no. ZDRCA2016003), Key Laboratory for Medicine of Jiangsu Province of China (no. ZDXKB2016005), and Jiangsu Province Capability Improvement Project through Science, Technology and Education (no. ZDXK202239), Young Scholars Fostering Fund of the First Affiliated Hospital of Nanjing Medical University (no. PY2022032), Special Fund project of Laboratory Medicine Research of Jiangsu Medical Association (no. SYH‐3201160‐0056).

## CONFLICT OF INTEREST STATEMENT

The authors declare that they have no conflict of interest.

## ETHICS STATEMENT

The studies involving human participants were reviewed and approved by the Ethics Committee of the First Affiliated Hospital of Nanjing Medical University (Nanjing, China) (Ethics review no: 2017‐SRFA‐064). The patients/participants provided their written informed consent to participate in this study.

## Data Availability

The data that support the findings of this study are available from the corresponding author upon reasonable request.
